# Effectiveness of long-lasting insecticidal nets in prevention of malaria among individuals visiting health centres in Ziway-Dugda District, Ethiopia: matched case–control study

**DOI:** 10.1186/s12936-021-03833-9

**Published:** 2021-07-03

**Authors:** Mesfin Kelkile Wubishet, Gebretsadik Berhe, Alefech Adissu, Mesfin Segni Tafa

**Affiliations:** 1 Ethiopian Field Epidemiology and Laboratory Training Programme (EFELTP), Arsi Zonal Health Department, Oromia, Ethiopia; 2 School of Public Health, College of Health Sciences, Mekelle University, Mekelle, Ethiopia; 3 Ethiopian Federal Ministry of Health, Addis Ababa, Ethiopia; 4 Epidemiology At Arsi Universities, Department of Public Health, College of Health Science, Asella, Ethiopia

**Keywords:** Effectiveness, Malaria, Matched case–control, Long-lasting insecticide-treated nets, Ziway-Dugda district, Ethiopia

## Abstract

**Background:**

Malaria is a major health problem in Ethiopia. Sleeping under long-lasting insecticidal nets (LLINs) is its major control strategy. Despite high LLINs use (84%) in Ziway-Dugda District, malaria remained a public health problem, raising concern on its effectiveness. Understanding the effectiveness of malaria control interventions is vital. This study evaluated the effectiveness of LLINs and determinants of malaria in Ziway-Dugda District, Arsi Zone Ethiopia.

**Methods:**

A matched case–control study was conducted among 284 study participants (71 cases and 213 controls) in Ziway-Dugda District, Arsi Zone, Ethiopia from March to May, 2017. Three health centers were selected randomly, and enrolled individuals newly diagnosed for malaria proportionally. Cases and controls were individuals testing positive and negative for malaria using rapid diagnostic tests. Each case was matched to three controls using the age of (5 years), gender and village of residence. The information was collected using pre-tested structured questionnaires through face to face interviews and observation. Data were entered into Epi-Info version 3.5, and analysed using Stata version-12*.* Conditional logistic regression was performed, and odds of LLINs use were compared using matched Adjusted Odds Ratio (AOR), 95% confidence interval (CI) and p-value of < 0.05.

**Results:**

One hundred twenty-three (61.2%) of the controls and 22 (32.8%) of cases had regularly slept under LLINs in the past two weeks. Using multivariate analysis, sleeping under LLINs for the past two weeks (AOR = 0.23, 95%CI = 0.11–0.45); living in houses sprayed with indoor residual spray (IRS) (AOR = 0.23, 95%CI: 0.10–0.52); and staying late outdoors at night in the past two-weeks (AOR = 2.99, 95%CI = 1.44–6.19) were determinant factors.

**Conclusions:**

Sleeping under LLINs is effective for malaria prevention in the district. IRS and staying late outdoors at night were determinants of malaria. It is recommended to increase attention on strengthening LLINs use and IRS in the area.

## Introduction

Malaria is a parasitic disease caused by Plasmodium species which is transmitted by the bite of female *Anopheles* mosquitoes [1, 2]. Sleeping under insecticide-treated nets (ITNs) is the most widely adopted and cost-effective preventive measure against malaria. It has long been used against mosquito bites and, effective because female mosquitoes transmitting malaria in most malaria-endemic regions only bite at night. It protects people at least by acting as a physical barrier; the chemical in it repels or kills mosquitoes and/or, through the ‘community effect’. The community effect occurs when the majority of people sleep under ITNs, resulting in an overall reduction of the mosquito population, thereby reducing the transmission. Most malaria-endemic countries have adopted policies to promote universal access to ITNs based on World Health Organization (WHO) recommendations. On this basis, over one billion nets have been distributed in Africa since 2000. As a result, the proportion of the population and under-five children sleeping under ITNs in sub-Saharan Africa has increased to 55% and 68%, respectively in 2015, resulting in a significant reduction of malaria cases. Of the total 663 million cases averted due to total malaria control interventions, 69% were due to ITNs utilization [3–6].

Key components of the malaria control programme are implemented by the Ethiopian Federal Ministry of Health (FMOH). The goals were set to be achieved through different strategies primarily emphasizing vector control activities. The major vector control activities implemented in the country include long-lasting insecticidal nets (LLINs), IRS, and environmental management. Universal LLNs coverage is promoted giving priority to pregnant women, and early treatment of clinical cases [5, 7, 8].

Currently, the country aimed to achieve universal coverage by distributing one LLIN per 1.8 persons through mass, free distribution campaigns at the community level. The objectives are to ensure 100% of households in malarious areas own at least one LLIN per sleeping space, and 80% of people at risk use it. On this basis, the FMOH has distributed 29.6 million LLINs through the campaign in 2015 [6, 7, 9, 10].

Despite a wide distribution and use of these nets as a major malaria control strategy, their current effectiveness against malaria has never been evaluated in the study area. Hence, evaluating the effectiveness of LLINs is important for prioritizing malaria control strategies in the area. Nearly half of the global population was at risk of malaria, and over half of all the countries are affected in 2015. A total of 214 million cases and 438,000 deaths of malaria occurred globally in 2015. The disease remained endemic in all the six WHO regions, the burden being heaviest in Africa. Over 90% of malaria cases and 92% of malaria deaths in 2015 occurred in Africa [1, 3, 11].

Despite the lower parasite prevalence compared to many African countries, malaria remained the major public health issue in Ethiopia, accounting for more than 60% of the population at risk (12). It was the leading cause of outpatient visits and admissions in the country during the past several years. According to the Federal Ministry of Health (FMOH), about 2.2 million cases and 662 deaths of malaria were reported on the country during 2014/15 [7, 10, 12, 13].

Malaria has placed a heavy economic burden in the country, as its peak transmission period coincides with the planting and harvesting seasons. The transmission is seasonal and unstable, largely determined by altitude and climate. It peaks bi-annually with the major transmission occurring between September and December, and the second “minor” transmission occurring between April and May. Due to this unstable transmission, protective immunity is relatively low in the country. Hence, unlike large parts of sub-Saharan Africa, all age groups are at risk [7–9, 12]. Malaria is also the leading communicable disease in Oromia, affecting 82% of woredas in the region [14].

Despite its progress, malaria has continued to be the major public health problem in Arsi Zone, becoming more difficult for its elimination. The prevalence of confirmed malaria cases was 16 per 10,000 populations in 2015/16 in the zone. Over 30% of the cases in the zone were contributed from Ziway-Dugda District during the year. Various publications indicated that malaria control should be focused on specific types of socio-demographic, housing, and environmental factors influencing its distribution in terms of seasonality and transmission intensity. Ownership and use of LLINs is the most commonly indicated factor influencing malaria morbidity and mortality worldwide [15–17]. Thus, it is a major malaria control strategy used in Ziway-Dugda District within the past ten years. However, despite its high coverage (84%) in the district, the disease remained to be a health problem in the area.

On the other hand, these days effectiveness of LLIN is being threatened by mosquito behavioural change and resistance of mosquito vectors to insecticides on the nets [18]. The resistance has been reported from different areas of the world including Ethiopia since 2010 [3, 19]. Hence, this development and spread of insecticide resistance could also raise concerns about the effectiveness of LLINs [7]. Therefore, the effectiveness of LLINs remained a question in the study area in the context of intensifying insecticide resistance in the country. Despite the use of LLINs, there was no study conducted in the area showing its effectiveness in the prevention of malaria. Therefore, this study was designed to evaluate the present effectiveness of LLINs and identify why the malaria burden persists even under high LLINs coverage in Ziway-Dugda District.

## Methods

### Study area and period

The study was conducted in the Ziway-Dugda District of Arsi Zone, Oromia, Ethiopia from March 1 to May 6, 2017. The district is located in the Great Rift-Valley at 222 kms distance East of Addis Ababa, the capital of Ethiopia. It is bordered in West and North by East-Shewa Zone, in South by Munesa, in East by Hitosa, and in South-East by Tiyo Districts. It is administratively divided into 30 kebeles (28 rural and two urban), with the district capital being at Ogolcho Town.

According to the population projection of the 2007 National Census, the district has a total population of 137,227 in 2016/17. Most (92%) of the population in the district are rural dwellers and almost half (51%) of them are females in 2016/17. Under-five children constitute 16.5% of the total population in the district. The climatic zone of the district is mainly Woina Dega. Its altitude ranges from 1500 to 2300 m above sea level. According to data obtained from the district administrative office, the estimated average annual temperature and rainfall of the district are 19.3 °c and 837 mm respectively.

The district has no hospital but has six health centres (HCs) and 30 health posts providing different health services including prevention and control of malaria. The physical primary health service coverage of the district is above 100%, with 242 health professionals in the district. Malaria is endemic in all kebeles of the district. By the year 2015/16, a total of 1289 cases were reported in the district. According to the district health office report, IRS and LLINs coverage of the district were 86% and 84% in 2015/16, respectively.

### Study design

A matched case–control study was conducted to assess the effectiveness of LLINs. These types of study designs have also been used in similar studies conducted previously in different countries to assess the effectiveness of their existing programmes [20–23].

### Source population

The source population was all individuals visiting all health centres in Ziway-Dugda District with the age of six months and above in 2017.

### Study population

The study populations were all individuals visiting Ongolcho, Cheffe-Jila, and Arata Health Centres in Ziway-Dugda District, above the age of six months.

### Eligibility

#### Inclusion criteria

They had lived within the catchment areas of the health centres for at least six months prior to the data collection period.

- They were newly diagnosed with positive or negative antigen Rapid Diagnostic Test (RDT) results respectively in the health centres during the study period.

#### Exclusion criteria

Individuals who had a history of malaria during the past six months prior to data collection, taken any of the anti-malarial treatments before coming to the health centres within the past two weeks and seriously ill during the data collection time were excluded from the study.

#### Matching criteria

Controls were individually matched with cases using age interval of (5) years, gender, and place of residence using Kebele/ village and/ or neighbourhood.

### Sample size determination

The sample size was calculated using Epi-info7stat-calc for an unmatched case–control study. During the calculation, an assumption of at least 70% protection conferred by using LLINs was taken in to account, from studies in Bungoma [24], and Cameroon [25]. In addition, the following assumptions were considered accordingly:

P1: 27.5% is the proportion of persons using LLINs among controls [26] (Table 1) 80% power, 95% confidence interval and 5% alpha error, Odds ratio of 0.3 [24, 25]. C: 3 is the number of controls per case (1:3 ratio).

**Table 1:** Socio-demographic characteristics of malaria cases and controls among individuals visiting health centres in Ziway-Dugda Distict, Ethiopia, 2017 (N = 268)

Variable	Category	Cases	Controls
		No. (%) n = 67	No. (%) n = 201
Age^a^	< 5	14 (20.9)	39 (19.4)
	5–14	17 (25.4)	51 (25.4)
	15–34	23 (34.3)	72 (35.8)
	35–49	7 (10.4)	22 (10.9)
	50 +	6 (9.0)	17 (8.5)
Sex^a^	Male	36 (53.7)	108 (53.7)
	Female	31 (46.3)	93 (46.3)
Residency^a^	Rural	47 (70.1)	141 (70.1)
	Urban	20 (29.9)	60 (29.9)
Religion	Muslim	38 (56.7)	112 (55.7)
	Orthodox	24 (35.8)	69 (34.3)
	Protestant	5 (7.5)	20 (10.0)
Marital status	Married	39 (58.2)	130 (64.7)
	Single	18 (26.9)	50 (24.9)
	Divorced or widowed	10 (14.9)	21 (10.4)
Educational level	No education	21 (31.3)	52 (25.9)
	Primary	24 (35.8)	69 (34.3)
	Secondary	17 (25.4)	60 (29.9)
	Higher education	5 (7.5)	20 (9.9)
Ocupational status	Farmer	17 (25.4)	50 (24.8)
	Student	17 (25.4)	49 (24.4)
	Housewife	13 (19.4)	45 (22.4)
	Merchant	9 (13.4)	27 (13.4)
	Daily laborer	7 (10.4)	15 (7.5)
	Employee	4 (6.0)	15 (7.5)
Estimated monthly Household income-quintiles (ETB)	Lowest 20% (< 501)	13 (19.4)	44 (21.9)
	Second (501–900)	18 (26.9)	37 (18.4)
	Middle (901–1400)	15 (22.4)	35 (17.4)
	Fourth (1401–2420)	9 ( 13.4)	44 (21.9)
	Highest (> 2420)	12 (17.9)	41 (20.4)
Family size	1–3	8 (11.9)	38 (18.9)
	4–6	48 (71.7)	130 (64.7)
	> 6	11 (16.4)	33 (16.4)
Number of under-five children	No children	28 (41.8)	94 (46.8)
	One children	30 (44.8)	78 (38.8)
	More than one children	9 (13.4)	29 (14.4)

^a^Matched variables

Using the above assumptions, the initial sample size became 256 (64 cases and 192 controls). By adding a non-response rate of 10%, the final sample size became 284 (71 cases and 213 controls). This provided 71 matched sets of case-controls (quadruplets).

### Sampling techniques and procedures

**Fig. 1:**
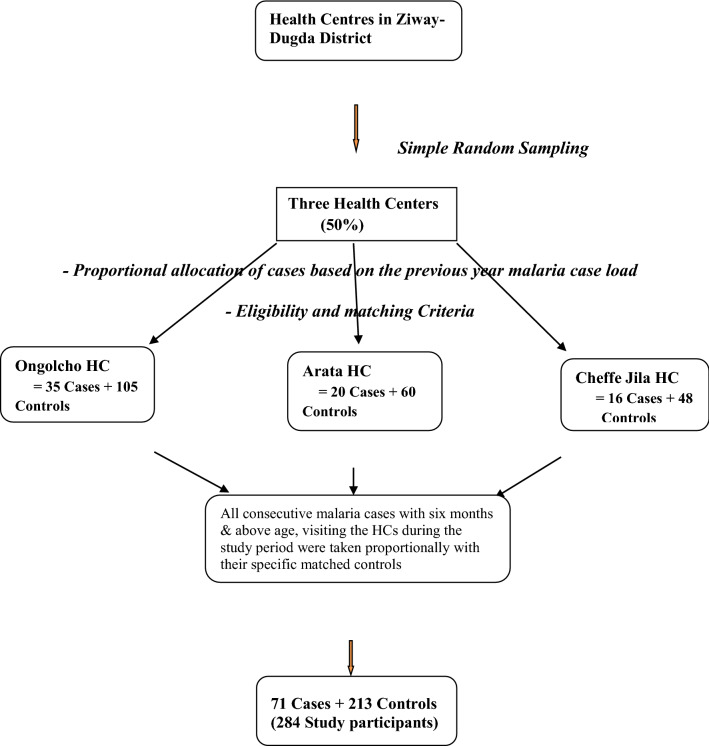
Sampling procedures followed during the selection of cases and controls among individuals visiting health centres in Ziway-Dugda Distict, Ethiopia, 2017

A simple random sampling method was used to select three of the six health centres in the district that gives 50%. Cases were individuals who were newly diagnosed with malaria positive RDT result in the selected health centres during the data collection period, and taken consecutively till the sample to be filled. However, the number of cases taken from each selected health centre was proportionally allocated based on the malaria caseload of the health centres during the previous year of the same months. Controls were then selected from individuals visiting the health centres where cases were drawn, using matching criteria with cases. This was to increase their comparability and minimize the possible effect of confounders. Each of the identified cases in the health centre was matched with three newly diagnosed individuals with negative RDT results (controls) and selected in similar manner as cases selected (Fig. 1).

### Study variables

#### Dependent variable

Malaria status.

#### Independent variables

The main predictor of interest taken to assess the effectiveness of LLINs was regularly sleeping under LLINs for the last two weeks before the onset of disease in cases. This was by considering the incubation period of *Plasmodium falciparum* (14 days) (26), which were the common species of malaria at the regional level in 2015 (12). Moreover, the following factors were taken as independent factors:

#### Socio-demographic factors

Age in years, sex, residency, religion, marital status, educational status, occupation, family size, number of children, and estimated household income.

#### Housing and related factors

Type of roofing, separated kitchen, separated bedroom, presence of windows, presence of patients with similar signs in-home, and house sprayed with IRS in the last 12 months.

### Knowledge and practices towards malaria prevention

#### Factors related to LLINs

LLINs ownership, regularly sleeping under LLINs for the last two weeks, sleeping under LLINs the previous night, number of LLINs in-home, age of LLINs (in months), presence of holes or tears in LLINs, frequency of washing LLINs in the last six months.

#### Environmental factors

Presence of stagnant water, living together with livestock’s, presence of forest or any vegetations near to home, presence of intermittent rivers or irrigations, and staying late outdoor during night.

### Data collection tools

Blood was collected from each study participant and examined using rapid diagnostic testing (RDT) for the presence or absence of malaria. Pre-tested structured questionnaires were used to collect data by interview and observation.

### Data collection procedures

A community sensitization campaign to increase health facility use for fever treatment was conducted during the study period with the purpose of obtaining an adequate number of cases. Then, informed written consent and/or assent were obtained from each individual coming to the health centres with febrile illness during the study period. Each volunteered individual was tested for malaria using RDT. Then, all individuals with positive RDT results were enrolled in the study as cases based on the illegibility criteria. Each case was matched with three controls from individuals visiting the health centres. After obtaining their informed written consent and/or assent, individuals tested with negative RDT results were enrolled in the study as controls with similar illegibility criteria as the cases.

Data on socio-demographic factors and LLINs use were then collected in the health centres for both cases and controls. Within the week of their health facility visit, the data collectors visited each case and control at their home, and collected data on housing conditions, environmental risk factors, and LLINs status.

### Data quality assurance

The questionnaires first prepared in English were translated into the local language, *Oromiffa*, and then re-translated back to English for consistency. Data was collected by six data collectors and two supervisors who were health workers in the district trained for two days on the purpose of the study, methods and procedures of the data collection. A pre-test was done using 5% of the sample size prior to the actual data collection in *Kiyansho* Health Centre. This was conducted by the principal investigator and two health staffs who were assigned as supervisors during the actual data collection and necessary modifications were done on the questionnaires. Supervision, follow-ups and appropriate corrections were done on daily basis during the data collection process by the supervisors. Prior to entering the data into the computer, the missing variables, consistency and its completeness were checked out and cleaned. Any error encountered was addressed immediately before the analysis, and data of two cases were discarded from analysis with their matched controls due to their poor quality. The data was entered into a computer by two persons to minimize error, and then checked using simple statistics such as frequency and sorting for consistency.

### Data management and analysis

The data was checked-up both at the field and after it was collected, for its completeness and consistency. Then, it was coded and entered into *Epi-Info version 3.5*, and cleaned. Then, the data was exported to both *SPSS version 20* and Stata *version* 12 for analysis. The descriptive analysis was performed using the *Epi-Info* database and *SPSS*, while the analytical part was performed using the *Stata.* The normality of continuous variables was checked using a histogram*.* Descriptive statistics were computed for continuous variables and reported with a mean (standard deviation (SD)) and median (inter-quartile range) for variables with approximately normal and non-normal distributions respectively. Categorical variables were reported by frequency and percentages. Proportions of each exposure variable among cases and controls were calculated. Using conditional logistic regression, variables associated with malaria with p-value < 0.05 in bivariate analysis were included in the multivariate analysis. The matched Adjusted Odds Ratio (AOR), and their corresponding 95% Confidence Interval (CI) and p-values were calculated to measure the significance of the association. Interpretations at p-value < 0.05 were done for statistical significance. The odds of regularly sleeping under LLINs among cases and controls were compared within the past two weeks*.* The effectiveness of LLINs was calculated as (1- the matched AOR) × 100. The multi-colinearity was checked for the independent variables. Hosmer and Lemeshow goodness of model fitness test was also conducted.

### Operational definitions

#### Case

A case was an individual who had a high axillary temperature (≥ 37.5 °C) or history of fever during 48 h proceeding the day of blood sampling, with a positive RDT result in one of the three health centres in Ziway-Dugda District (20, 26, 27).

#### Control

A control was an individual fulfilling matching criteria with the case, who had no fever or signs suggesting malaria within the past two weeks, with a negative RDT result in one of the three health centres in Ziway-Dugda District (26).

#### LLIN users

In this study, LLIN use was taken in two ways:

Individuals reported regularly sleeping under LLIN within the past two-weeks from the onset of fever or other malaria symptoms in cases (14 out of 14 days) [20, 26, 27]. Individual reported sleeping under LLIN the previous night [7, 12]

#### Non-LLIN users

Individuals were classified as non-LLIN users if one of the following met:

- If the household of the individual had no LLIN during the home visit or.
- If LLIN was available in the household, but the individual had not used it at all or.
- If LLIN was available in the household, but either the individual had not regularly slept under it within the past two weeks or did not slept under it the previous night.

#### LLINs ownership

Households having one or more LLINs during the home visit [7, 12]

#### Effectiveness of LLINs

Effectiveness of LLINs was calculated as (1- AOR) × 100; where AOR is the matched adjusted odds ratio of LLINs use among cases and controls, using its corresponding 95% CI and p-values of < 0.05 for statistical significance test [21, 26].

#### Knowledge

Knowledge was measured based on the ability of each respondent to correctly respond to ten questions regarding malaria. Each question contains one point for the correct or positive responses, and 0 for wrong or negative responses and the total response contains 10 points. The total response score was then classified into three levels according to Bloom’s cut-off point, 60%–80%, adopting from other studies [28, 29]:

- High-level: knowledge score that fell eight and above points (80%–100%).
- Moderate-level: knowledge score that fell 6–7.9 points (60%–79%).
- Low-level: knowledge score that fell below six points (less than 60%).

#### Practice

It was measured based on the respondent’s previous health-seeking behaviour, decisions and actions taken towards the prevention and control of malaria using a total of five questions. Each question contains one point for positive or correct lifestyle practice, 0.5 for neutral lifestyles and 0 points for negative or wrong life style practices. The total response score was five points and classified into three according to Bloom’s cut-off point (60%–80%), adopting from other studies [28, 29]:

- **Good**: practice score that fell four and above points (80%–100%).
- **Fair**: practice score that fell 3–3.9 points (60%–79%)
- **Poor**: practice score that fell below three points (less than 60%).

### Ethical considerations

Ethical approval was secured from the Institutional Review Board of Mekelle University College of Health Sciences School of Public Health. Then, further consent obtained from Oromia Regional Health Bureau, Arsi Zonal Health Department and Zuway district health office based on the hierarchy of the authority of the offices. Written informed consent was obtained from each study participant or children’s parent after explaining them the purpose and objectives of the study. For study participants younger than 18 years, the consent was taken from parents; written assent was also obtained from participants aged 12 to17 years. Confidentiality of the information each participant given were respected. Appropriate measures were taken to assure confidentiality of the information. After the interview, health education was given for each respondent on malaria transmission and its preventive measures. Individuals diagnosed with positive malaria test result were immediately treated with ant-malarial drugs free-of-charge.

## Results

### Socio-demographic characteristics

In this case–control study, 71 matched sets of case-controls (284 individuals) were identified. Two (2.8%) of the cases were not incorporated into the study due to the inaccessibility of their home. In addition, data of two (2.8%) cases were discarded with their matched controls due to their poor quality. Finally, data of 67 matched sets of case-controls (268 individual participants) were included in the analysis with a response rate of 94.4%.

Age ranges from six months to 70 years, with a median of 16 years (IQR: 6–29) in cases and 16 years (IQR: 6–30) in controls. Family size ranges from two to ten with the average members of 5.1 (SD 1.4) in cases and five (SD 1.6) in controls per household. More than half of both, the cases and controls (53.7%) were Male. More than two-thirds of both the cases and controls (70.1%) were rural residents. Slightly above two-thirds (68.7%) of the cases and slightly below three-fourth (74.1%) of controls had attained at least primary education (Table 1).

### Housing and environmental factors related to malaria prevention

More than half (55.2%) of the cases and 126 (62.7%) of the controls live in houses made of corrugated iron sheet roofing. The presence of diseased family members with similar signs were reported in almost one-fourth (25.4%) of the cases and 26 (12.9%) of controls. More than half (52.2%) of the cases and 141 (70.1%) of the controls live in houses sprayed with IRS during the past twelve months Thirty-one (62%) cases and 106 (57.6%) of controls owning LLINs at their home had two or more LLINs (Table 2).

**Table 2:** Housing condition, environmental and LLINs related factors to malaria prevention among cases and controls in individuals visiting health centres in Ziway-Dugda Distict, Ethiopia, 2017 (N = 68)

Variable	Category	Cases	Controls
		No. (%) n = 67	No. (%) n = 201
Type of roofing	Corrugated iron sheet	37 (55.2)	126 (62.7)
	Thatched roof	30 (44.8)	75 (37.3)
Separated bedroom	No	34 (50.7)	96 (47.8)
	Yes	33 (49.3)	105 (52.2)
Separated kitchen	No	24 (35.8)	82 (40.8)
	Yes	43 (64.2)	119 (59.2)
Presence of windows	No	29 (43.3)	78 (38.8)
	Yes	38 (56.7)	123 (61.2)
Presence of patients with similar signs in home	No	50 (74.6)	175 (87.1)
	Yes	17 (25.4)	26 (12.9)
House sprayed with IRS	No	32 (47.8)	60 (29.9)
	Yes	35 (52.2)	141 (70.1)
Presence of stagnant water	No	58 (86.6)	165 (82.1)
	Yes	9 (13.4)	36 (17.9)
Living together with livestock’s	No	47 (70.1)	155 (77.1)
	Yes	20 (29.9)	46 (22.9)
Presence of forest or vegetation near to home	No	58 (86.6)	178 (88.6)
	Yes	9 (13.4)	23 (11.4)
Presence of intermittent rivers or irrigation	No	55 (82.1)	162 (80.6)
	Yes	12 (17.9)	39 (19.4)
Staying late out-door during night in the past two weeks	No	37 (55.2)	137 (68.2)
	Yes	30 (44.8)	64 (31.8)
Number of LLNs in home	Only one	19 (38.0)	78 (42.4)
	Two or more	31 (62.0)	106 (57.6)
Age of LLINs in months	≤ 12	28 (56.0)	116 (63.0)
	13–35	18 (36.0)	58 (31.5)
	> 35	4 (8.0)	10 (5.5)
Presence of holes in LLINs	No	36 (72.0)	129 (70.1)
	Yes	14 (28.0)	55 (29.9)
Frequency of washing LLINs in the past six months	< 2 times	42 (84.0)	140 (76.1)
	≥ 2 times	8 (16.0)	44 (23.9)

### Knowledge and practices towards malaria prevention

**Fig. 2:**
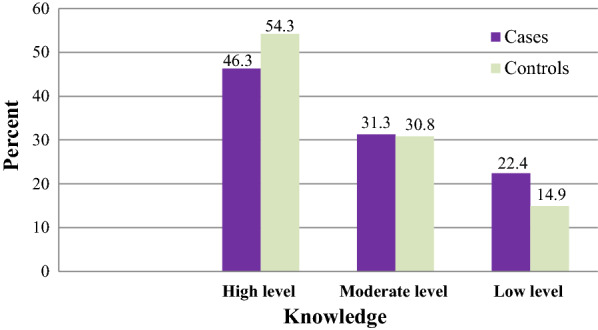
Malaria cases and controls by their score of knowledge towards malaria prevention among individuals visiting health centres in Ziway-Dugda Distict, Ethiopia, 2017 (N = 268)

**Fig. 3:**
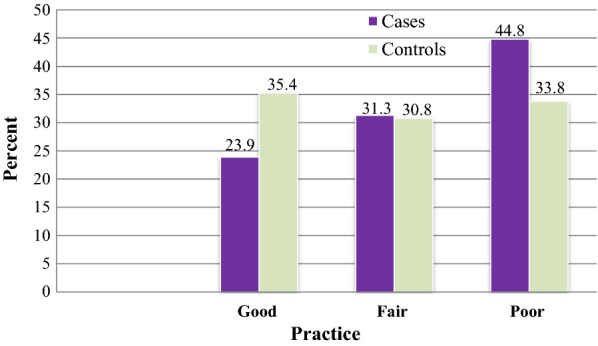
Malaria cases and controls by their score of practices towards malaria prevention among individuals visiting health centres in Ziway-Dugda Distict, Ethiopia, 2017 (N = 268)

Thirty-one (46.3%) cases and more than half (54.3%) of the controls had a high-level knowledge of malaria prevention (Fig. 2). Only slightly below one-fourth (23.9%) of the cases and 71 (35.4%) of the controls had good practices towards malaria prevention (Fig. 3).

### Ownership, utilization and status of LLINs

**Fig. 4:**
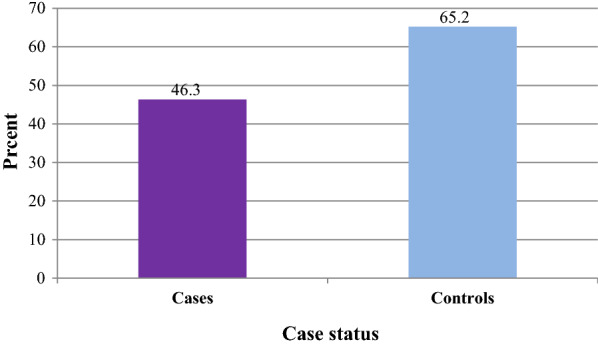
Proportion of malaria cases and controls slept under LLINs in the previous night among individuals visiting health centres in Ziway-Dugda Distict, Ethiopia, 2017 (N = 268)

**Fig. 5:**
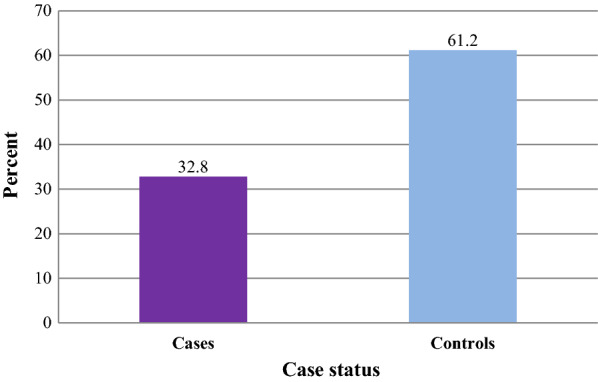
Proportion of malaria cases and controls regularly slept under LLINs for the last two weeks among individuals visiting health centres in Ziway-Dugda Distict, Ethiopia, 2017 (N = 268)

The presence of at least one LLINs in the home was observed in nearly three-fourths (74.6%) of the cases and 91.5% of controls. Similarly, both sleeping under LLINs in the previous night and regularly sleeping under LLINs for the last two weeks were lower among the cases than in controls (Figs. 4 and 5).

### Effectiveness of LLINs and determinants of malaria prevention

Using bi-variable conditional logistic regression analysis, the presence of LLINs in-home, sleeping under LLINs in the previous night, and regularly sleeping under LLINs for the past two weeks were significantly associated with malaria. In addition, the presence of patients with similar signs in home, staying late outdoor during the night in the past two weeks, poor practices related to malaria prevention compared to good practices, and living in houses sprayed with IRS during the past 12 months were significantly associated with the disease (Table 3).

**Table 3:** The bi-variate conditional logistic regression of factors related to malaria prevention among cases and controls in individuals visiting health centres in Ziway-Dugda Distict, Ethiopia, 2017 (N = 228)

Variables	Category	Cases	Controls	Crude Matched Odds Ratio (95% CI)
		No. (%) n = 67	No. (%) n = 201	
Educational level	No education	21 (31.4)	52 (25.9)	1
	Primary	24 (35.8)	69 (34.3)	0.81 (0.38, 1.74)
	Secondary and above	22 (32.8)	80 (39.8)	0.58 (0.25, 1.33)
Type of roof	Corrugated iron sheet	37 (55.2)	126 (62.7)	0.64 (0.33, 1.26)
	Thatched roof	30 (44.8)	75 (37.3)	1
Presence of windows	No	29 (43.3)	78 (38.8)	1
	Yes	38 (56.7)	123 (61.2)	0.79 (0.42, 1.49)
Presence of patients with similar signs in home	No	50 (74.6)	175 (87.1)	1
	Yes	17 (25.4)	26 (12.9)	2.22 (1.13, 4.38)*
House sprayed with IRS	No	32 (47.8)	60 (29.9)	1
	Yes	35 (52.2)	141 (70.1)	0.34 (0.17, 0.67)**
Knowledge related to malaria prevention	High-level	31 (46.3)	109 (54.2)	1
	Moderate-level	21 (31.3)	62 (30.9)	1.25 (0.64, 2.44)
	Low-level	15 (22.4)	30 (14.9)	2.15 (0.90, 5.13)
Practice related to malaria prevention	Good	16 (23.9)	71 (35.3)	1
	Fair	21 (31.3)	62 (30.9)	1.61 (0.74, 3.48)
	Poor	30 (44.8)	68 (33.8)	2.25 (1.04, 4.85)*
Presence of LLINs in home	No	17 (25.4)	17 (8.5)	1
	Yes	50 (74.6)	184 (91.5)	0.27 (0.13, 0.58)**
Sleeping under LLINs the previous night	No	36 (53.7)	70 (34.8)	1
	Yes	31 (46.3)	131 (65.2)	0.43 (0.24, 0.76)**
Regularly sleeping under LLINs for the last 2 weeks	No	45 (67.2)	78 (38.8)	1
	Yes	22 (32.8)	123 (61.2)	0.28 (0.15, 0.52)***
Living together with livestock’s	No	47 (70.1)	155 (77.1)	1
	Yes	20 (29.9)	46 (22.9)	1.61 (0.79, 3.31)
Presence of intermittent rivers or irrigation	No	55 (82.1)	162 (80.6)	1
	Yes	12 (17.9)	39 (19.4)	0.89 (0.39, 2.00)
Staying late out-door during night within the past 2 weeks	No	37 (55.2)	137 (68.2)	1
	Yes	30 (44.8)	64 (31.8)	1.95 (1.04, 3.65)*

Significance: * Significant (p < 0.05);* * strongly significant (p < 0.01); ***highly significant (p < 0.001)

After adjusting for all variables significant in bivariate conditional logistic regression, cases had 77% lower odds of sleeping regularly under LLINs than controls for the past two weeks (AOR: 0.23, 95% CI: 0.11, 0.45). Similarly, the odds of living in houses sprayed with IRS during the past twelve months was 77% lower among the cases than in controls (AOR: 0.23, 95% CI: 0.10, 0.52). On the other hand, cases were nearly three times more likely to stay late outdoor during night time in the past six months than controls (AOR: 2.99, 95% CI: 1.44, 6.19) (Table 4).

**Table 4:** The summary of multi-variate conditional logistic regression of independent factors associated with malaria prevention among cases and controls in individuals visiting health centres in Ziway-Dugda Distict, Ethiopia, 2017 (N = 268)

Factors	Category	Crude matched odds ratio (95%CI)	Adjusted matched odds ratio (95% CI)
Presence of patients with similar signs in home	No	1	1
	Yes	2.22 (1.13, 4.38)	1.91 (0.86, 4.26)
House sprayed with IRS	No	1	1
	Yes	0.34 (0.17, 0.67)	0.23 (0.10, 0.52)***
Practice related to malaria prevention	Good	1	1
	Fair	1.61 (0.74, 3.48)	1.05 (0.42, 2.58)
	Poor	2.25 (1.04, 4.85)	0.85 (0.33, 2.18)
Presence of LLINs in home	No	1	1
	Yes	0.27 (0.13, 0.58)	0.52 (0.21, 1.31)
Sleeping under LLINs the previous night	No	1	
	Yes	0.43 (0.24, 0.76)	1.40 (0.42, 4.66)
Regularly sleeping under LLINs for the last 2 weeks	No	1	1
	Yes	0.28 (0.15, 0.52)	0.23 (0.11, 0.45)***
Staying late out-door during night in the past two weeks	No	1	
	Yes	1.95 (1.04, 3.65)	2.99 (1.44, 6.19)**

Significance: *Significant (p < 0.05); **strongly significant (p < 0.01); ***highly significant (p < 0.001)

## Discussion

This study assessed the effectiveness of LLINs in the prevention of malaria. The result of the study shows a higher proportion of controls (65.2%) than the cases (46.3%) had slept under LLINs in the previous night. About (61.2%) of the controls and (32.8%) of cases had regularly slept under LLINs for the past two weeks. Using multivariate conditional logistic regression, cases were 77% times less likely to regularly sleep under LLINs for the past two weeks than the controls. In the same manner, cases were 77% times less likely to live in houses sprayed with indoor residual spray during the past twelve months than the controls. On the other hand, cases were nearly three times more likely to stay late outdoor at night in the past two weeks than the controls in the study area.

### Effectiveness of LLINs in prevention of malaria

The study showed that regularly sleeping under LLINs for the past two weeks among the study participants had protected 77% of individuals using it from malaria. This individual protectiveness of LLINs is supported with results of similar studies around the World. LLINs were reported to have at least a 50% protective effect from malaria across different studies [30]. A case–control study in Benin had shown its protection of up to 55% against malaria in children [26]. A case–control study in South West Cameroon had also shown a 70% protection of LLINs use against malaria [25]. Similarly, a cohort study conducted in Malawi had indicated that the use of LLINs had reduced significantly the incidence of malaria in children by 30% [15]. The individual-level protectiveness of LLINs in this study results is also supported with a case–control study in Bungoma that shown regularly sleeping under LLINs had conferred 77% protection against malaria [24].

In a similar way, a retrospective cohort study in the country, Aletawondo Woreda had also shown that regularly sleeping under LLINs had reduced the incidence of malaria by 43.5% [31]. Besides, this results on the effectiveness of LLINs is also supported by a study on the resistance of mosquitoes to insecticides on LLINs in the country that revealed the effectiveness of these nets for at least 32 months [32]. Despite the variation in the exact figure of LLINs protectiveness across these studies, all of them had indicated that LLINs are still effective in the prevention of malaria supporting these results. However, the variations in the exact figure of its protectiveness could be due to two aspects related to methodologies used across the studies: One might be due to the variation in study designs used, and the other could be due to variation in the population studied across the studies.

On contrary, this study results was not consistent with studies indicating the non-effectiveness of LLINs currently on malaria prevention. Both case–control studies conducted in Machinga District of Malawi in 2013 [27] and Haiti in 2012 [20], and a cluster-randomized controlled trial conducted in Rakhine State Western Myanmar in 2013 [33] had all revealed that LLINs did not significantly protect against malaria. As these areas are highly endemic for malaria, people may develop higher protective immunity as a result of long exposure to malaria parasites. Therefore, the short-term effect of LLINs in the area might not be clearly seen. In addition, asymptomatic Plasmodium infections are more common in highly endemic areas than in areas of low endemicity. It is also more common in the most immune compared to those who have not had exposure long enough to develop immunity [34]. Hence, these conditions could underestimate the short-term protective effect of LLINs in the areas. Effectiveness of LLINs in the areas could also be compromised due to the insecticide resistance of mosquito vectors. This might occur as a result of the high coverage of LLINs and IRS in the areas as the major malaria control strategies. This causes longer exposure of mosquito vectors to the chemicals within LLINs and IRS, thereby increasing insecticide resistance [4, 18].

On the other hand, the higher individual-level protective effect seen in this finding could be due to lower community-wide protective effects of LLINs in the study area as a result of lower coverage of LLINs use. Studies had shown that LLINs confer protection on community members even that may not sleep under a net [35]. This community-wide effect is achieved when the majority of people sleep under LLINs, resulting in an overall reduction of indoor resting and feeding mosquitoes, thereby reducing the transmission [3–5]. Hence, to achieve the maximal effectiveness of nets, its coverage must be high and individuals should properly deploy their nets each night [6]. However, the result of this study shows that only 60% and 54% of the visited households had used LLINs the previous night and regularly slept under it for the last two weeks, respectively. Therefore, this lower utilization of LLINs could lower its community-wide protectiveness. During this case, only individuals sleeping under LLINs could be protected from malaria leaving other members of the community without protection. This condition could exaggerate the individual level protectiveness of LLINs as most cases may come from households with no LLINs. Moreover, taking a short duration of study period which was not on the season of high-level malaria occurrence in the country for evaluating LLINs programme could also inflate its effectiveness in our study.

On the other hand, the nets are expected to be more effective in areas where the major vectors bite primarily indoors or late at night than where the vectors feed outdoors before people go to sleep, or after they get up [36]. This means, ITNs are more targeted to indoor resting (*endophilic*) and indoor biting (*endophagic*) mosquitoes species. The former mosquito behavioral pattern is expected in Ziway-Dugda District which might also explain the high individual protective effect of LLINs observed in the area.

### Determinants of malaria prevention

The study indicated that living in houses sprayed with IRS during the past twelve months was associated with the lower odds of the disease. This result was consistent with both studies conducted in Omeya District, Oromia in 2012 [37], and Haiti in 2012 [20], both of which were indicating the decreased odds of malaria among individuals living in houses sprayed with IRS. This may suggest the effectiveness of the current insecticides within IRS in reducing malaria among the users.

The present study has also indicated that staying late outdoor during the night in the past two weeks was significantly associated with the higher odds of malaria. This result is supported by studies conducted in Tanzania and Cambodia both of which had indicated a higher outdoor malaria transmission where the bites had occurred before the sleeping time [38, 39]. This could be due to the increased risk of exposure to outdoor feeding Anopheles mosquitoes carrying Plasmodium specious during staying late out-door at night time [38]. This occurs as people in rural communities could stay late outside the home during night-time particularly in dry and hot seasons due to different socio-economic purposes, and sharing sociocultural ceremonies or grieves. This issue calls for the need of focusing on outdoor malaria preventive strategies such as environmental management and wearing personal protective clothes in addition to strategies targeted to indoor feeding mosquitoes (IRS and LLIN) in rural areas.

On the other hand, the study did not find any evidence of association with socio-demographic factors with malaria. However, various works of literatures showed that malaria is influenced by a wide range of socio-demographic factors [27, 40, 41]. Nevertheless, despite the rest factors which did not showed a significant association in our study, variables such as age, gender, and place of residence were excluded from the analysis as a result of matching to minimize their possible confounding effect. Similarly, this study did not indicate any evidence of association that shows housing characteristics such as type of roof, the presence of separated kitchen, separated bedroom and presence of windows to be either risk or protective factor of malaria. However, studies had shown that these housing characteristics are predictors of malaria [42].

Despite their lack of association in this study, many studies have also indicated that environmental risk factors are also predictors of malaria. For instance, the presence of forests near house and plants used for fencing [41], and living in areas where stagnant water or irrigation existed [16, 43–45] were all shown to be associated with greater odds of the disease in other studies. Lack of association of these factors in the present study could be attributed due to factors like the presence of stagnant water, forests near home, intermittent rivers and living together with livestock’s were lower both among the cases and controls.

## Strengths and limitations

### Strengths

To assess the effectiveness of LLINs, case–control study provide useful alternatives to prospective and experimental studies as the former study types avoid most of the ethical and coast issues related to the later studies [26, 46, 47]. In addition, cohort studies are not-practical in low-transmission settings because large sample sizes are required for diseases with low occurrence as malaria is mainly seasonal unlike case–control study. Moreover, it is impossible to find groups with LLINs coverage and groups without LLINs coverage in the population of our situation for comparison (either using cohort or randomized control trials). In such situations, case control study is less expensive and operationally feasible alternatives. This study has also other strengths in that possible confounding variables were matched to minimize their effect of confounding on the outcome, thereby to increase precision and strength of evidence in the study. Matching could also increase the comparability of cases and controls. On the other hand, the primary objective in this study was to assess the effectiveness of LLINs. However, due to the convenient nature of case–control study to assess multi-exposure factors for a single disease, the determinants of malaria also assessed in the area. This was also done with the intention of overcoming the effect of possible confounders. Taking only confirmed cases and controls to reduce misclassification bias could also be the other strength of this study. Regardless of identifying both the cases and controls from health centres for the sake of obtaining adequate number of cases, both the cases and controls were taken from the same community, matching them by place of residence. This could also increase the comparability of cases and controls.

## Limitations

Regardless of the above strengths, this study also lacks many issues. This case–control study is expected to provide a lower strength of evidence compared to prospective and experimental studies. On the other hand, due to the drawback of matching, possible confounding variables such as age, gender, and place of residence were not considered in the analysis. Therefore, the effect of these variables on the outcome was not evaluated in this study. This study could also be subjected to different types of biases. For instance, it might be subjected to recall bias because most of the presence of exposure ascertainment was done through respondents' information. Misclassification bias might also be a threat for this study because the reported regular users might be irregular users. This means, individuals may reply as being regular users of LLINs without being the actual regular users as a result of their social desirability feeling. Moreover, a short duration of study period which was not on the national expected season of high-level of occurrence of malaria could inflate the effectiveness of LLINs in the study area. The other limitations of this study were the type of LLINs underuse was not identified and the occurrence of relapse cases is obviously unrelated to ITN use so the fact that the analysis fails to differentiate between P. falciparum and P. vivax cases.

## Conclusions and recommendations

This study has showed that regularly sleeping under LLINs for the past two weeks has significantly protected individuals of six months and above from malaria in the area. This provides adequate evidence that regular use of LLINs is still effective in prevention of malaria among individuals visiting health centres in the study area. In addition, this study has indicated that living in houses sprayed with indoor residual spray during the past twelve months and staying late outdoor during night were determinants of malaria prevention in the area.

Regular use of LLINs should be strengthened in the study area. It is important supply LLINs continuously and conducting indoor residual spray regularly. The district health office, health centres and health posts should conduct community sensitization campaign to increase their strict use of control measures such as regular use of LLINs, IRS and personal protective. Further study, whether LLINs are equally effective for malaria prevention during high transmission seasons, and whether factors like age, gender and place of residence will affect malaria prevention.

## Data Availability

Additional detailed information and raw data will be shared upon request addressed to the corresponding author.

## Abbreviations

AOR: Adjusted odds ratio
CI: Confidence interval
COR: Crude odds ratio
E.C.: Ethiopian calendar
ETB: Ethiopian birr
FMOH: Federal ministry of health
HC: Health center
IQR: Inter-quartile range
IRS: Indoor residual spray
ITNs: Insecticide-treated nets
LLINs: Long-lasting insecticide-treated nets
MIS: Malaria indicator survey
NMSP: National malaria strategic plan
OR: Odds ratio
ORHB: Oromia regional health bureau
RDT: Rapid diagnostic test
RWI: Household relative wealth index
SD: Standard deviation
SPH: School of public health
SPSS: Statistical package for social science
WHO: World Health Organization
